# The gut microbiome in tuberculosis susceptibility and treatment response: guilty or not guilty?

**DOI:** 10.1007/s00018-019-03370-4

**Published:** 2019-11-15

**Authors:** Osagie A. Eribo, Nelita du Plessis, Mumin Ozturk, Reto Guler, Gerhard Walzl, Novel N. Chegou

**Affiliations:** 1 DST-NRF Centre of Excellence for Biomedical Tuberculosis Research, South African Medical Research Council Centre for Tuberculosis Research, Division of Molecular Biology and Human Genetics, Faculty of Medicine and Health Sciences, Stellenbosch University, P.O. Box 241, Cape Town, 8000 South Africa; 2 International Centre for Genetic Engineering and Biotechnology (ICGEB), Cape Town-Component, Cape Town, 7925 South Africa; 3 Institute of Infectious Diseases and Molecular Medicine (IDM), Department of Pathology, Division of Immunology and South African Medical Research Council (SAMRC) Immunology of Infectious Diseases, Faculty of Health Sciences, University of Cape Town, Cape Town, 7925 South Africa

**Keywords:** Tuberculosis therapy, Mycobacterium tuberculosis, Gut commensal, Microbial imbalance, Immune response, Microbiome biosignatures

## Abstract

Although tuberculosis (TB) is a curable disease, it remains the foremost cause of death from a single pathogen. Globally, approximately 1.6 million people died of TB in 2017. Many predisposing factors related to host immunity, genetics and the environment have been linked to TB. However, recent evidence suggests a relationship between dysbiosis in the gut microbiome and TB disease development. The underlying mechanism(s) whereby dysbiosis in the gut microbiota may impact the different stages in TB disease progression, are, however, not fully explained. In the wake of recently emerging literature, the gut microbiome could represent a potential modifiable host factor to improve TB immunity and treatment response. Herein, we summarize early data detailing (1) possible association between gut microbiome dysbiosis and TB (2) the potential for the use of microbiota biosignatures to discriminate active TB disease from healthy individuals (3) the adverse effect of protracted anti-TB antibiotics treatment on gut microbiota balance, and possible link to increased susceptibility to *Mycobacterium tuberculosis* re-infection or TB recrudescence following successful cure. We also discuss immune pathways whereby the gut microbiome could impact TB disease and serve as target for clinical manipulation.

## Introduction

Tuberculosis (TB) is an infectious disease caused by the non-motile, acid fast bacillus *Mycobacterium tuberculosis* (*M.tb*). TB is spread when infectious aerosol droplets containing the bacilli are released from an infected individual, typically through sneezing or coughing [1]. Although only 5–10% of the estimated 1.7 billion people infected with *M.tb* will progress to active TB disease during their lifetime, approximately 1.6 million people died of the disease in 2017 alone [1]. TB is currently ranked as the foremost cause of death from a single pathogen. Several underlying immune, environmental and host genetic predisposing factors have been associated with TB including diabetes, infection with HIV, malnutrition and deficiency in interferon-gamma (IFN-γ) encoding genes [1]. However, one emerging host factor that may be associated with TB disease is the gut microbiota (microbial community inhabiting the gut) [2, 3]. It is known that at birth, the gut becomes colonized by commensal microbes that make up the gut microbiota. These gut microbes closely interact with components of the immune system and accordingly, the composition and metabolic activities of these gut bacterial networks shape and participate in the development and proper functioning of both adaptive and innate immunity [4]. Typically, these interactions between the microbiota and immune system are homeostatic and tightly regulated. Therefore, any disturbance in this finely turned balance could influence host immunity [4]. Recent literature has linked dysbiosis (a state of microbial imbalance) in microbiota community to compromised immune protection against *M.tb* infection, leading to increased susceptibility or recurrence of TB disease [2, 3]. In this review, we summarize emerging data describing the association between the gut microbiome and lung immunity during TB disease. We also discuss possible mechanisms by which the gut microbiota may impact TB immunity and/or treatment response and outcome.

## The gut microbiome composition is altered during TB disease and anti-TB drug treatment

Many studies investigating perturbations in the gut microbiome during TB disease and the profound effect of anti-TB drug therapy on the gut microbiome composition are currently emerging. A recent study reported a decline in the alpha diversity of the gut microbiome after pulmonary *M.tb* infection. However, these alterations were minimal and were mainly observed in the comparative abundance of species within the genus *Bacteroides* [5]. In contrast, many species from the genus *Bacteroides* increased in abundance during anti-TB antibiotics treatment, including *Bacteroides fragilis*, whereas, the population of members within the Clostridiales order, declined significantly [5]. An earlier study suggests that the overall microbiome diversity during TB drug therapy does not differ from those of uninfected humans [6]. However, substantial decline in specific gut microbiota taxa was reported in individuals undergoing anti-TB antibiotics treatment compared to both latently infected and uninfected humans [6]. Individuals on anti-TB drug therapy had an enrichment of *Erysipelatoclostridium*, *Fusobacterium* and *Prevotella*, whereas, depletion of *Blautia*, *Lactobacillus, Coprococcus*, *Ruminococcus* and *Bifidobacterium* was observed in comparison to the latent TB group. Furthermore, after more than 1 year of stopping treatment, the intestinal microbiome of the individuals cured of TB (through 6 months anti-TB drug treatment), was clearly distinguishable from the latent TB cohorts, indicating that treatment for TB has a long-lasting effect on microbiome composition [6]. A similar study investigated this outcome using mouse model [7]. The result showed that infection of mice with H37Rv *M.tb* strain caused distinct changes in the diversity of the gut microbiome especially in the order Clostridiales. Furthermore, many genera within the class *Clostridia* such as *Ruminococcus, Butyricicoccus, Acetivibrio, Alkaliphilus* and *Peptococcus* declined in their relative population during treatment. Interestingly, only the gut composition of members of the genus *Erysipelatoclostridium* increased during treatment [7].

In another study, the gut microbiome composition of individuals presenting with recurrent TB (previously declared as cured) contrasted with those of healthy controls [8]. Microbiota within the phylum Bacteroidetes were depleted in recurrent TB cohorts when compared with healthy individuals. On the contrary, the population of members of the phyla Actinobacteria and Proteobacteria, containing numerous diseases causing bacterial species was increased in recurrent TB cases. Furthermore, compared to healthy individuals, there was a decline in the population of the genus *Lachnospira* and *Prevotella* in individuals newly diagnosed with active TB and in those presenting with recurrent TB [8]. The authors reasoned that preserving a normal and balanced composition of gut microbiome, could play a crucial role in the prevention of TB recurrence and in host recovery from the disease [8]. These reports bring to the fore the yet unanswered questions namely; (1) are alterations in the gut microbiome a cause or consequence of immune dysfunction attributable to disease states such as TB? (2) are anti-TB drugs alone sufficient to treat the disease, to enable sterilizing cure, at least in all patients? This is important given recent findings that patients who had successfully undergone standard TB treatment and were clinically cured still had positron emission tomography-computed tomography (PET/CT) imaging patterns that were consistent with active TB. Furthermore, *M.tb* mRNA was detected in bronchoalveolar lavage and sputum samples collected from these patients, with TB disease recurring in some of the patients within 2 years from treatment completion and presumed cure [9].

## Pre-treatment with narrow spectrum anti-TB antibiotics impairs alveolar macrophage metabolism and function

Although anti-TB antibiotics are effective in killing *M.tb*, recent literature have taken into account the profound gut microbiome dysbiosis induced by anti-TB drug therapy [6–8]. Whereas isoniazid, ethambutol and pyrazinamide purportedly have a narrow spectrum activity against mycobacteria, rifampicin has a broad-spectrum effect [10]. A worrisome outcome of this anti-TB drug-induced gut microbiome perturbation is the possibility of increasing susceptibility to subsequent re-infection or recrudescence of TB disease after being cured. More so, a study by Verver et al. [11] which showed that the prevalence rate of TB ascribable to re-infection after successful treatment was four times that of new TB cases, gives credence to this possibility. However, studies investigating this potential adverse effect of anti-TB antibiotics on the immune response to *M.tb* are scarce.

In a recent study, Khan et al. [3] began to address this critical knowledge gap by investigating why host immune system fails to generate permanent protection against *M.tb* despite protracted anti-TB antibiotics treatment. The study showed that treating mice with a combination of isoniazid and pyrazinamide or rifampicin alone, significantly altered the gut microbiome. Isoniazid/pyrazinamide treatment expanded the abundance of Bacteroidetes. Whereas, rifampicin depleted Firmicutes population while increasing the abundance of Verrucomicrobia and Bacteroidetes phyla. At the genus, differences in *Clostridia* IV and XIV clusters were the most noteworthy change in the isoniazid/pyrazinamide-treated animals. Interestingly, dysbiosis in gut microbiome resulting from treating these mice with isoniazid/pyrazinamide as opposed to rifampicin led to an increase in *M.tb* load [3]. Furthermore, this effect (increased susceptibility) was reversed by faecal microbiome transplantation from untreated mice. Functionally, impairment of alveolar macrophage metabolism concomitant with defective bactericidal activity was linked to the increased susceptibility of the isoniazide/pyrazinamide-treated animals [3]. Alveolar macrophages isolated from isoniazide/pyrazinamide-treated animals displayed dampened spare respiratory capacity, basal respiration and ATP production and were more tolerant to *M.tb* growth. In addition, the production of interleukin (IL)-1-beta (β) and tumor necrosis factor-alpha (TNF-α), together with the expression of major histocompatibility complex class II (MHCII) significantly declined after *M.tb* infection [3].

Another striking finding from the study was that adoptive transfer of *M.tb*-infected alveolar macrophages from the isoniazid/pyrazinamide-treated animals significantly increased *M.tb* load in recipient mice [3]. How this anti-TB-drug-induced dysbiosis alters alveolar macrophage function is presently unknown. However, the authors speculated that changes in peripheral circulation of metabolites produced by gut microbiota following isoniazid/pyrazinamide treatment could possibly have influenced alveolar macrophage metabolism [3, 12]. Altogether, the study suggests that narrow-spectrum anti-TB antibiotics has profound effect on the gut microbiome which in turn negatively impacts macrophage immune defense against *M.tb.* By interpreting these data we could infer that upon successful TB treatment and cure (1) gut microbiome community is perturbed (2) this gut microbiota dysbiosis impact negatively on macrophage metabolism (3) as a result macrophage mycobactericidal activity is impaired upon subsequently *M.tb* infectious challenge, leading to successful re-infection (4) balance in gut microbiome composition is vital to sustain alveolar macrophage response against *M.tb*. However, studies detailing these associations are only emerging and would require further validation. Future studies could investigate the involvement of other functional and phenotypic immune markers. In addition, such studies may include compositional and functional analysis of gut microbiota metabolites, e.g., short chain fatty acids (SCFAs) in peripheral circulation during anti-TB antibiotics treatment.

## Gut microbiota signatures distinguish active TB patients from healthy individuals

Recently, there has been an intensified search for biomarker signatures that could accurately diagnose TB, predict progression from latent to active TB, assist in monitoring the response to anti-TB therapy and prediction of treatment outcome. In the wake of emerging literature on gut microbiota dysbiosis associated with TB and anti-TB drug treatment, developing gut microbiota biosignatures for TB disease and treatment response could be a promising area for investigation. Hu et al. [13] in a recent report profiled the gut microbiota community of patients with pulmonary TB versus healthy controls and identified significant changes in the microbiota composition and associated metabolic pathways. Differential abundance of 25 microbiota was identified between the TB and control cohorts. Two bacterial species were enriched in TB patients, whereas 23 were abundant in healthy controls. Among the bacterial species that were abundant in the control cohorts, nine were gut microbiota that produce SCFAs such as propionate, butyrate, acetate and lactate. They include; *Ruminococcus obeum, Bifidobacterium longum, Roseburia intestinalis, Roseburia inulinivorans, Coprococcus comes, Akkermansia muciniphila, Eubacterium rectale, Bifidobacterium adolescentis* and *Roseburia hominis* [13]. In addition, ascorbate and biotin biosynthesis were abundant in healthy controls, whereas flavin, folate, vitamin B6 and thiamine biosynthetic pathways were conspicuous in TB patients.

Besides strengthening the integrity of intestinal epithelial cells, SCFAs play an important role in inflammatory responses in the gut and at distal mucosal sites such as the respiratory tract [14, 15]. Many cells express G protein-coupled receptors (GPCRs) such as GPR41, GPR43 and GPR109A, and SCFAs activate host immunity by interacting with these receptors [16]. In this way, SCFA can induce either pro- or anti-inflammatory responses depending on the signal transduction pathway. For example, GPR41 and GPR43 signaling can commit to mitogen-activated protein kinases (MAPK) activation thereby inducing a pro-inflammatory response. On the other hand, GPR43 can activate β-arrestin-2 activation pathway resulting in an anti-inflammatory milieu through the inhibition of nuclear factor kappa-light-chain-enhancer of activated B cells (NF- κB) [17]. This underscores the significance of a homeostatic environment composed of different SCFAs that induces both pro- and anti-inflammatory responses. In addition, butyrate stimulates the secretion of IL-10 from dendritic cells and macrophages in the gut by signaling through GPR109A [18, 19]. SCFAs also promote the expansion of regulatory T cells (Treg) particularly along the gut–lung axis through the inhibition of histone deacetylase [15, 20]. Therefore, increase in systemic inflammation and concomitant impairment of immune responses in TB patients may imply loss of microbiota that produce SCFAs [13]. Meanwhile, accumulating evidence suggests that type 2 diabetes (T2D) is associated with a decrease in the abundance of SCFA producers [21]. T2D poses a significantly increased risk for the development of active TB [22]. It is possible that gut microbiome dysbiosis involving SCFA producers could represent a link between T2D and TB. Hence, an improved understanding of this hypothetical microbiome-mediated causal relationship between T2D and TB is imperative.

Notably, Hu et al.’s [13] study demonstrated the potential for the use of microbiota biosignatures for the diagnosis or discrimination of active TB cases from health individuals. Three microbiota biosignature comprising of *Roseburia hominis*, *Roseburia inulinivorans* and *Hemophilus parainfluenzae* were selected after five repeated experiments and cross validation using a training set consisting of 31 healthy controls and 30 TB patients [13]. The area under curve (AUC) when using these three bacterial species for discriminating active TB from healthy individuals was 84.6%. An independent test set consisting of 16 TB patients and 30 healthy controls likewise indicated that the model performs well with an AUC of 76.7% [13]. In addition, analysis of metagenome-wide single nucleotide polymorphisms (SNPs) for *Bacteroides vulgatus* identified 46 SNPs that were differentially distributed between the two groups. In a related earlier study, an increase in gut microbiota that produces butyrate was reported in TB patients when compared to close household contact as healthy controls [23]. These gut bacteria include *Eubacterium rectale, Faecalibacterium prausnitzii*, and *Roseburia inulinivorans* [23]. Taken together, these studies underpin the likely involvement of SCFAs and their pathways in TB and the possibility of developing gut microbiota biosignatures that delineate the disease stages. Nevertheless, more detailed metabolomic studies involving larger participant sizes from different geographical settings and designed to include the different transition points in the life cycle of TB disease are needed.

## Gut microbiota regulates immune cell phenotypes/*Mycobacterium tuberculosis*-induced immune responses

Commensal microbiota regulates both adaptive and innate immunity directly or indirectly by producing small molecules (metabolites) which influence the threshold of immune activation following pathogen stimulations. In line with this role, although epithelial cell barrier supposedly restricts microbes to the gut, microbial metabolites can infiltrate epithelial cell boundary. These metabolites then aggregate in host circulation and are sensed by circulating immune cells [24]. Therefore, the release of metabolites by gut microbiome species rather than the direct communication between gut bacteria and immune cells is more likely to modulate host immune defense during disease. In addition to providing signals for immune cells, these metabolites also exert direct microbicidal effect on pathogens. For example, the gut microbiota *Clostridium sporogenes* produce indole-3-propionic acid (IPA) from the metabolism of tryptophan. IPA readily percolates gut barrier and accumulate in human circulation [25]. In one study, IPA reduced *M.tb* burden in a mouse model and the molecule was well tolerated showing adequate pharmacokinetic properties [26]. Although the mechanism by which IPA exerts this effect is still under investigation, preliminary evidence suggests that IPA mirrors tryptophan, the physiological allosteric inhibitor of the enzyme (anthranilate synthase), which catalyzes the primary step in tryptophan biosynthesis. Consequently, regardless of intracellular tryptophan levels, IPA switches off tryptophan production in *M.tb* [27].

Indeed, specific gut microbiota species have been shown to induce different immunological phenotypes or cytokine responses, which may influence disease pathogenesis or pathology [28]. For example, the expansion of CD4 + T cells was shown to be induced in germ-free mice colonized with *Bacteroides fragilis* strains that produced polysaccharide-A (PSA). This CD4 + T cell proliferation restored balance between Th (T helper)-1 versus Th2 cytokines by increasing IFN-γ and TNF-α production in the germ-free mice [29]. Similarly, an increase in IL-10 secretion was associated with enhanced anti-inflammatory signaling from both systemic and intestinal Treg in gnotobiotic mice colonized with a cocktail of mouse-derived *Clostridia* strains [30].

In addition to innate immune responses, elimination or control of *M.tb* requires a coordinated and balanced expression of pro-and anti- inflammatory T cell subsets and regulatory T cell phenotypes. Early evidence suggests that the gut microbiota may be critical for maintaining this balance. For instance, Dumas et al. [2] reported that increase in pulmonary colonization by *M.tb* was prompted by antibiotics-induced alterations in the diversity of the gut microbiome. On one hand, there was no substantial change in the recruitment of neutrophils, macrophages, and dendritic cells to the lungs between the untreated and antibiotics-treated mice. Furthermore, production of the pro-inflammatory cytokines, IFN-γ, TNF-α and IL-1β, remained unchanged in the antibiotics-treated animals [2]. However, a decrease in the number of mucosal associated invariant T (MAIT) cells; a lymphocyte population with characteristics resembling innate cells, in the lungs, was observed in the microbiome-altered animals. This effect on MAIT cells was linked with the diminished ability of these animals to resist *M.tb* infection [2]. Additionally, there was a decline in IL-17A production by MAIT cells, with the decline in MAIT cells’ proliferating ability upturned after faecal microbiome transplantation in the antibiotics-treated mice.

IL-17 secretion is associated with increased recruitment of neutrophils, and optimal Th1 cell inflammatory responses [31, 32]. IL-17 is also required for adequate T cell localization within lymphoid follicles in the lungs, an event which promotes efficient macrophage activation and early protective immune response against *M.tb* [33]. In addition, IL-17 was shown to inhibit the development of hypoxic and necrotic granulomas, thereby limiting TB disease severity [34]. The role of IL-17 during vaccine-induced immunity against *M.tb* is also increasingly being recognized [35–37]. Dumas and colleagues reasoned that enhancing the functions of MAIT cells may represent one probable mechanism by which the gut microbiota contribute to protection against *M.tb* colonization [2].

In a similar study, antibiotic-induced changes in the gut diversity of *M.tb*-infected animals compromised mouse immunity and increased the ability of the pathogen to spread to other organs [38]. This disruption in the gut microbiota was shown to modify the adaptive cell-mediated immune responses to *M.tb*, with Tregs expanding in numbers while IFN-γ and TNF-α- producing Th1 cells diminished in their frequencies. Strikingly, after fecal transplant, TB immunity was reestablished and the spread of *M.tb* to different organs was prevented [38]. In a human study that evaluated the interaction of inflammatory biomarkers with the gut microbiome in people with active and latent TB infections prior to anti-TB treatment, Firmicutes/Bacteroidetes ratio correlated to the levels of measurable IL-1β in TB disease [39]. The number of neutrophils in peripheral blood was correlated to the relative abundance of Bacteroidetes in latent and active TB, whereas the comparative plenitudes of Coriobacteriales was positively correlated to IFN-γ production in latent TB cases [39]. The authors concluded that in the active TB cases, low Firmicutes/Bacteroidetes proportion and gut dysbiosis with higher comparative abundances of Bacteroidetes in stool correlates to systemic proinflammation, whereas in latent TB, a dose–response relationship between the comparative abundance of Bacteroidetes and peripheral polymorphonuclear neutrophils persists but does not prompt systemic inflammation [39].

Neutrophils form an integral part of the early immune response to *M.tb* and granuloma formation, although they play a controversial role during TB disease. While some studies associate the abundance of neutrophils to protection against TB, others have suggested that disease progression is associated with the accumulation of neutrophils [40–42]. It is assumed that during the early stages of TB, the abundance of neutrophils is protective, whereas, at the later stages, they may be associated with unfavorable outcomes. In a study by Martineau and colleagues, the risk of developing TB disease in close contacts of TB patients was inversely related to the number of circulating neutrophils in peripheral blood [40]. In the same study, depletion of peripheral neutrophils reduced the ability of blood cells to inhibit the growth of *M.tb* and *M. bovis* BCG [40]. Sugawara and colleagues also showed that increasing the number of circulating neutrophils in rats through LPS stimulation reduced pulmonary *M.tb* CFUs following infection [43]. Furthermore, neutrophils recovered from these animals were mycobactericidal [43]. Nevertheless, whether microbiota-driven changes in circulating neutrophils have any direct impact on their role in *M.tb* resistance is a question that requires further investigation.

Another consideration is that immune cells mounting a challenge against infection by *M.tb*, are pre-polarized by responses generated against other infections, including gut microbiota-associated infections. For example, infection by *Helicobacter hepaticus* significantly influenced TB subunit-vaccine-induced protection through an IL-10 dependent pathway [44]. *H. hepaticus* infection increased colonic IL-10 mRNA expression and mice susceptibility following *M.tb* challenge [44]. In addition, human adenovirus type 5 immunization of *H. hepaticus*-infected mice resulted in reduced protection against *M.tb.* Nevertheless, the protective impact of the subunit vaccine was reestablished following treatment with anti-IL-10 receptor antibody [44]. In a similar report, it was observed that individuals harbouring *Helicobacter pylori* infection were less likely to progress from latent to active TB when compared to *H. pylori* seronegative individuals. This was due to enhanced Th1 responses to TB antigens, and the outcome was the same even in individuals concurrently harbouring helminth infections [45]. This impact was speculated to be because of the collaboration between infections that modifies Th1 responses in addition to the reciprocal regulatory pathways prompted in individuals with high burden of infectious disease [45]. Reports of this nature emphasize the need for additional studies investigating the mutualistic or pathogenic interactions between *Helicobacter* species and the immune response in the gut. Important questions arising from these studies include; (1) how an unhealthy gut microbiome could be manipulated to restore its positive immune-response modulating effects on TB immunity, (2) the specific pathways implicated in the translation of the immune responses generated in the gut to protective lung immunity, (3) which specific microbiome species or cocktail of gut microbiota promote the expansion of immune cell phenotypes with specific roles in limiting TB disease. A summary of recent literature on gut microbiome and TB is provided in Table 1.

**Table 1:** Summary of recent studies on gut microbiome and tuberculosis

Authors	Study location	Study type	Study description	Main differences in microbiota between groups	Immune correlates/effect	Ref
Hu et al. (2019)	China	Human	A total of 46 TB cases and 61 controls. Patients were newly diagnosed with pulmonary TB and anti-TB drug naïve.	1. SCFA producers enriched in control cohorts compared to TB cases 2. Three microbiota signatures comprising *Roseburia hominis*, *Roseburia inulinivorans* and *Hemophilus parainfluenzae* discriminated active TB cases from healthy controls with AUC of 84.4%		[13]
Khan et al. (2019)	Canada	Animal	Experimental animals were pretreated with INH/PYZ or RIF followed by H37Rv infection. Control animals were antibiotics untreated but infected with H37Rv. 4-5 mice per group	1. Significant difference in *Clostridia* IV and XIV following INH/PYZ treatment. 2. RIF depleted Firmicutes population and increased *Verrucomicrobia* and Bacteroidetes abundance	INH/PYZ treatment dampened alveolar macrophage spare respiratory capacity, basal respiration and ATP production. Reduced IL-1β, TNF-α and MHCII. Increased macrophage permissiveness and mouse susceptibility to *M.tb* which was reversed by FT	[3]
Hu et al. (2019)	China	Human	A total of 61 TB cases, 10 LTBI and 13 healthy controls. TB cases were divided into 28 active TB, 13 and 10 TB patients on 1- and 2-weeks anti-TB therapy, respectively, and 10 cured TB patients	1. Minor changes during *M.tb* infection mainly within the genus *Bacteroides* 2. Decreased *Ruminococcus* and *Fecaelibacterium* belonging to *Clostridiales* and increased *Bacteroides* during anti-TB drug treatment.		[5]
Dumas et al. (2018)	France	Animal	Treatment of mice with broad-spectrum antibiotics, infection with *M.tb* followed by FT	*Bacteroidetes* and *Firmicutes* depleted, Proteobacteria enriched in antibiotics-treated animals	No change in neutrophils, macrophages, dendritic cells, IFN-γ, TNF-α and IL-1β in antibiotics-treated animals. Decrease in MAIT cells and IL17A in treated animals and increased *M.tb* susceptibility. FT improved immunity	[2]
Luo et al. (2017)	China	Human	37 TB patients and 20 healthy controls. TB patients were divided into NTB (new diagnose with TB and less than I week anti-TB treatment) and RTB (previously treated and cured prior to becoming culture-positive)	1. *Bacteroidetes* decreased while Actinobacteria and *Proteobacteria* increased in RTB 2. Depleted *Lachnospira* and *Prevotella* genus in NTB and RTB	Both *Prevotella* and *Lachnospira* positively and negatively correlated with CD4 T cell count in NTB and RTB, respectively.	[8]
Wipperman et al. (2017)	Haiti	Human	Cohorts of 19 TB patients on treatment, 19 patients treated and cured of TB and 75 controls. 3 TB patients were on treatment for more than 6 months. Controls were divided into 50 IGRA positive and 25 IGRA negative (LTBI)	1. *Clostridium*, *Fusobacterium* and *Prevotella* enriched whereas, *Lactobacillus, Coprococcus*, *Ruminococcus* and *Bifidobacterium* depleted in treatment cases 2. *Bacteroides* depleted, while *Ruminococcus, Faecalibacterium and Eubacterium* were enriched in cured patients		[6]

Authors	Study location	Study type	Study description	Key microbiota differences between groups	Immune correlates/effect	Ref
Huang et al. (2019)	Taiwan	Human	Cohorts consisting of 25 active TB, 32 LTBI and 23 healthy controls	Differences in *Firmicutes*/*Bacteroidetes* ratio between groups	Number of neutrophils correlated with abundance of *Bacteroidet*es in latent and active TB, *Coriobacteriales* abundance positively correlated to IFN-γ production in latent TB cases	[39]
Namasivayam et al. (2017)	USA	Animal	Infection of mice with *M.tb* followed by treatment with anti-TB drugs for up to 4 months	Anti-TB antibiotics*-*treated *depleted Ruminococcus, Butyricicoccus, Acetivibrio, Alkaliphilus and Peptococcus genera* while *Erysipelatoclostridium* was increased		[7]
Khan et al. (2016)	India	Animal	Treatment of mice with broad-spectrum antibiotics, infection with *M.tb* followed by FT	Decline in *Bifidobacterium*, *Lactobacillus Campylobacter* and *Bacteroides.* Increase in *Enterococcus* in treated animals	Increased Tregs, decreased IFN-γ and TNF-α- producing Th1 cells. Increased *M.tb* burden in lung and spleen in antibiotics-treated animals. FT improved mouse immunity	[38]
Maji et al. (2018)	India	Human	6 TB patients and 6 healthy household contacts as control. Stool collected from TB patients before the start of treatment, 1 week and 1 month into treatment.	1. Butyrate and propionate producing bacteria, e.g., *Eubacterium rectale, Faecalibacterium prausnitzii*, and *Roseburia inulinivorans* abundance increased in TB cases. *Prevotella* and *Bifidobacterium* were enriched in controls 2. Biosynthesis of amino acids and vitamin metabolism declined in TB patients		[23]

*SCFA* Short chain fatty acid, *FT* faecal transplant, *INH* Isoniazid, *RIF* Rifampicin, *PYZ* pyrazinamide, *LTBI* Latent tuberculosis infection, *IGRA* interferon gamma release assay, *IFN-γ* interferon gamma, *TNF-α* tumor necrosis factor-alpha, *Th* T helper, *IL*  interleukin, *MAIT* mucosal associated invariant T, *MHCII* Major histocompatibility complex II, *AUC* Area under curve

## Toll-like receptor signaling and immune cell homing along the gut–pulmonary axis

Bacterial peptidoglycan, polysaccharide, lipoteichoic acid and lipopolysaccharide (LPS) are known to stimulate toll-like receptor (TLR) signaling [46]. In addition, bacterial metabolites often find their way into the lymphatic system linking the gut–lung axis [47–49]. This bidirectional movement of metabolites could trigger innate immune cell activation such as macrophages and neutrophils [50] which are central in the elimination or control of *M.tb* infection [51]. Furthermore, lymphocytes express specific chemokine and adhesion receptors which enable them to be trafficked into tissues expressing their corresponding cognate ligands [52]. For example, dendritic cells (DCs) enhance the expression of chemokine receptor 4 (CCR4) on T cells which enables already polarized T cells to home into the lungs expressing increased levels of chemokine ligand 17 (CCL17) [53]. A study by Ichinohe et al. [54] showed that a single dose of LPS delivered intrarectally, restored lung immune responses of mice infected with influenza virus, mainly through gut-initiated TLR signaling pathway. A similar report corroborated this link between gut bacteria and lung immunity. In this study, depletion of *Bifidobacterium* and *Lactobacillus* with neomycin was associated with altered immune response to influenza A virus infection with concomitant increase in lung damage in a mouse model [55]. This antibiotic-induced dysbiosis inhibited TLR7 signaling, the event of which reduced the secretion of the downstream pro-inflammatory cytokines IFN-γ and IL-17, with a simultaneous increase in the levels of IL-4 and IL-10. However, after *Bifidobacterium* probiotic reconstitution of the gut microbiota, TLR7 response improved and restored the production of IFN-γ and IL-17 but remarkably inhibited IL-4 and IL-10 induction [55]. Lung damage was also reduced [55]. These data plainly suggest the involvement of TLR activation in immune crosstalk along the gut–lung axis. Understanding and maintaining this communication along the gut-pulmonary axis are especially important considering emerging literature linking gut microbiome dysbiosis and TB disease.

**Fig. 1:**
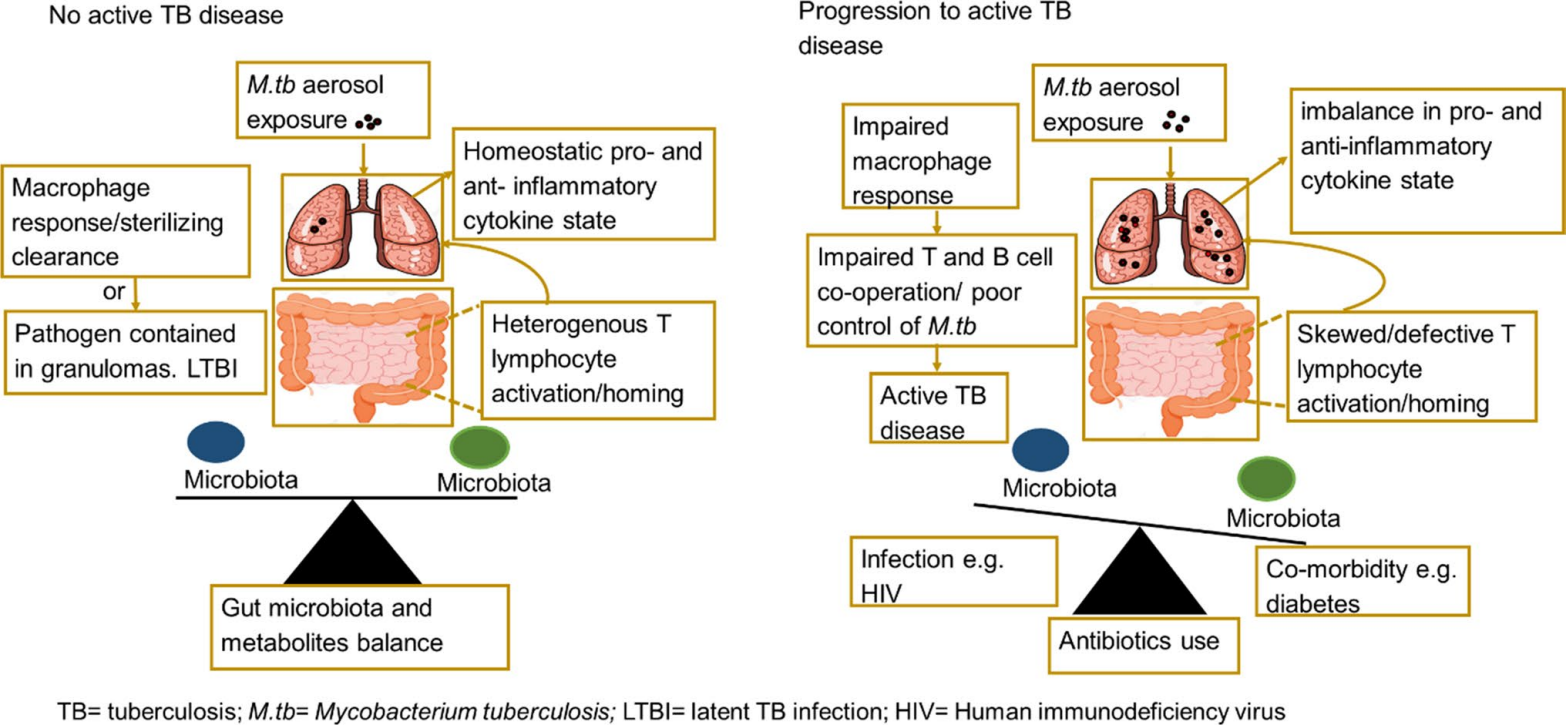
Model for gut microbiome and metabolite regulation of cytokine responses during tuberculosis disease. Heterogeneity and balance in gut microbiota and metabolites provide different signals that educate the immune system. Exposure to *M.tb* infection triggers gut–lung homing of pro- and anti-inflammatory T lymphocytes. Homeostatic cytokine lung environment is maintained. Macrophages clear infection or contain pathogen within granulomas in people shown as No TB disease. By contrast, factors such as antibiotics use, HIV infection and diabetes alter microbiota balance leading to defective/skewed T lymphocytes activation. *M.tb* infectious challenge in this state triggers over-abundance of a T lymphocyte subsets upon gut–lung homing. Macrophage response is impaired. Defective T and B cell cooperation results in poor control of infection, promotes infection of adjacent lung tissues and progression to active TB disease

In the case of *M.tb* infection, we could hypothesize that in a microbiota balance state, different gut commensal bacteria and metabolites provide signals that educate innate and adaptive immune cells while inducing both pro- and anti-inflammatory cell types. This implies that in addition to local immune defense, immune signals generated by gut microbiota will contribute to the pool of lymphocytes recruited to the airways upon *M.tb* infectious challenge. Therefore, heterogeneity in the immune response ensures a homeostatic lung cytokine environment (Fig. 1). This balanced “immune state” may lead to two possible outcomes (1) sterilizing clearance by innate responses whereby the exposed individual remain tuberculin skin test (TST) or interferon gamma release assay (IGRA) negative or (2) T and B cell cooperates, macrophages are activated to clear infection or contain the pathogen within granulomas leading to latent TB infection (LTBI). The integrity of granulomas is also maintained, as a result progression to active TB disease is prevented.

By comparison, the constant use of broad-spectrum antibiotics, for example to treat other infections may result in loss of beneficial microbiota thereby altering metabolite balance (Fig. 1). In addition, HIV infection and TB co-morbidity such as T2D alter microbiota community balance [56, 57]. HIV infection may prompt loss of interaction with CD4 + T cells that produce regulatory responses promoting the tolerance of beneficial microbiota. It can also result in the selection of inflammation-tolerant versus inflammation-sensitive gut microbiota due to chronic gut inflammatory state [56]. T2D on the other hand, is reported to deplete SCFAs producing microbiota [57]. These alterations in gut microbiota and metabolite composition may lead to (1) defective or skewed T lymphocytes activation (2) over-abundance of a T lymphocyte subset in the lungs creates an imbalance in pro- and anti-inflammatory cytokine state or (3) may give rise to heightened and dysregulated Th1 and Th17 responses as has been reported in TB-T2D [58, 59] (Fig. 1). Consequently, innate immune responses are impaired, there is also defective T and B cell cooperation and impairment of granuloma formation. Poor control of infection promotes escape of *M.tb* from granulomas, infection of adjacent lung tissues and progression to active TB disease (Fig. 1). However, detailed investigations are required to establish these relationships.

Therefore, studies aimed at unraveling which gut microbiota species or metabolites are necessary to sustain a normal gut microbiome-TLR signaling cascade and validation of gut-initiated T cell homing during *M.tb* infectious challenge will be a novel area for investigation. In addition, studies involving *M.tb* infection models of altered gut microbiome aimed at reconstituting the gut with specific gut microbiome species or cocktail of gut microbiota, may prove innovative for the identification of gut bacterial species whose immunomodulatory roles could positively impact TB immunity or limit disease severity.

## Gut microbiota and potential impact on TB drug pharmacokinetics: the role of probiotics

Gut microbiota play a role in the pharmacokinetics (PK) of drugs. Although the synthesis of primary bile acids and metabolism of drugs essentially occurs in the liver, secondary bile acids are mostly produced by the gut microbiota [60]. In addition, there is evidence supporting the role of the gut microbiome in modifying the expression levels of transporters and enzymes that metabolize drugs [60]. Gut microbiota could impact the bioavailability, efficacy and toxicity of drugs through different mechanisms such as: (1) producing drug activating or inactivating enzymes; for example, the conversion of sulfalazine to its active derivative, 5- amino 5-salicyclic acid by enzymes produced by gut microbiota [61] (2) binding directly to drugs thereby impacting their bioavailability; for instance, the bioavailability of l-3,4-dihydroxyphenylalanine (L- DOPA) is altered by binding of *H. pylori* [62, 63].

One common cause of treatment failure during TB therapy is the selection of resistant *M.tb* strains resulting from exposure to lower than therapeutic dose [64, 65]. Wide fluctuations in the PK of ethambutol, Isoniazid and pyrazinamide have also been reported in plasma [66, 67]. Among other factors, these fluctuations were accounted for by variables such as malnutrition, age, HIV and antiretroviral treatment [68, 69]. Interestingly, a number of these factors also impact on gut microbiome composition. Therefore, it is possible that their effect on anti-TB drug metabolism is indirectly linked to the alterations they induce on the microbiome. Apart from this, a more devastating outcome would be that fluctuations in anti-TB drug concentrations in plasma are a direct consequence of gut microbiome dysbiosis induced by the anti-TB drugs themselves. This possibility cannot be ruled out given recent data on the profound gut microbiome dysbiosis induced by anti-TB drugs [6, 7]. In the event of this possibility, could probiotics supplementation aimed at reconstituting the gut microbiome during anti-TB antibiotics treatment improve TB drug PK and consequently treatment outcome? Future studies could (1) measure drug PK in *M.tb*-infected mice treated with anti-TB antibiotics while simultaneously receiving faecal transplant from normal mice, and compare with antibiotics-treatment only controls (2) compare anti-TB drug PK in *M.tb-*infected germ-free mice vs conventionally colonized mice.

In the case of the cancer-targeting drug ipilimumab (a human monoclonal antibody targeting CTLA-4), the effectiveness of the drug was shown to be reliant on specific *Bacteroides* species [70]. In this report, germ-free and antibiotic-treated mice which were non-responsive to ipilimumab, were overturned by *B. fragilis* gavage, inoculation with *B. fragilis* polysaccharides, or by adoptive immunotherapy with *B. fragilis*-specific murine T cells [70]. This underscores the significance of a microbiota composition dominated by Bacteroidales during ipilimumab treatment. On the contrary, anti-PD-1 blockade treatment was not effective in patients with high comparative richness of *Bacteroides thetaiotaomicron,* while *Faecalibacterium* and Clostridiales enriched gut microbiota-favoured treatment efficacy [71]. Similarly, a mixture of *Bifidobacterium* and anti-PD-L1 monoclonal antibody treatment improved tumor control in mice when compared to the immunotherapeutic intervention alone in another study [72]. Likewise, a study conducted on human kidney transplantation patients suggests that gut microbiota could impact on the PK of the immunosuppressive drug tacrolimus [73]. As the drug has a narrow therapeutic spectrum, patients require monitoring to make certain that the optimum therapeutic dose is reached. Investigation of the gut microbiota community profile in patients reaching high doses of tacrolimus showed an abundance of *Faecalibacterium prausnitzii* [73]. *Faecalibacterium prausnitzii* is a butyrate-producing microbiota. Accordingly, the authors opined that tacrolimus drug metabolism could be connected to butyrate availability. These reports demonstrate that commensal microbiota could be manipulated for clinical advantage. This methodology could be explored during TB treatment using probiotics directed not at cure, but to dampen the effect of anti-TB antibiotics on gut microbiota community.

## Conclusion and future perspectives

Reports investigating whether alterations in the gut microbiome contribute to bias in inter-individual levels of susceptibility to *M.tb* infection or response to TB drug treatment are still emerging. Equally important is establishing whether gut microbiome dysbiosis induced by the protracted anti-TB antibiotics treatment is linked to increased susceptibility to *M.tb* re-infection or TB recrudescence after successful cure. This could change the way TB disease is currently treated and may translate into the development of new therapeutic approaches. Future directions may include:

1. Development of microbiota signatures that discriminate between the different stages in the life cycle of TB disease. Such studies may include a large cohort of participants from different geographical settings.
2. Investigating the impact of alterations in specific gut microbiota species on TB susceptibility and the immune cells/mechanisms involved.
3. Metabolomic and functional characterization of peripheral pool of metabolites produced by gut microbiota during the different stages of TB disease.
4. More studies investigating anti-TB drug-induced gut microbiome dysbiosis and the potential impact on susceptibility to re-infection, together with the associated immune cells and pathways affected.
5. Establishing whether dysbiosis induced by anti-TB drugs themselves following protracted use impact on the drug PK.
6. Developing animal models to explore whether anti-TB antibiotics treatment combined with probiotics (composed of specific microbial species or microbiota cocktail) will improve treatment response and outcome.

## Abbreviations

TB: Tuberculosis
*M.tb*: *Mycobacterium tuberculosis*
IFN-γ: Interferon-gamma
PET/CT: Positron emission tomography-computed tomography
IL-1β: Interleukin-1-beta
IL: Interleukin
TNF-α: Tumor necrosis factor-alpha
MHCII: Major histocompatibility complex class II
SCFAs: Short chain fatty acids
GPCRs: G protein-coupled receptors
MAPK: Mitogen-activated protein kinases
NF- κB: Nuclear factor kappa-light-chain-enhancer of activated B cells
Treg: Regulatory T cells
T2D: Type 2 diabetes
AUC: Area under curve
SNPs: Single nucleotide polymorphisms
IPA: Indole-3-propionic acid
CD: Cluster of differentiation
Th: T helper
MAIT: Mucosal associated invariant T
FT: Faecal transplant
INH: Isoniazid
RIF: Rifampicin
PYZ: Pyrazinamide
LTBI: Latent TB infection
NTB: New TB
RTB: Recurrent TB
TLR: Toll like receptor
DCs: Dendritic cells
CCR: Chemokine receptor
CCL: Chemokine ligand 17
LPS: Lipopolyssaharide
IGRA: Interferon gamma release assay
TST: Tuberculin skin test
PK: Pharmacokinetics
L-DOPA: L-3,4-dihydroxyphenylalanine
mRNA: Messenger ribonucleic acid
CFU: Colony forming unit
BCG: Bacillus Calmette-Guérin
